# Barriers to and facilitators of the implementation of multi-disciplinary care pathways in primary care: a systematic review

**DOI:** 10.1186/s12875-020-01179-w

**Published:** 2020-06-19

**Authors:** Eva Seckler, Verena Regauer, Thomas Rotter, Petra Bauer, Martin Müller

**Affiliations:** 1 Centre for Research, Development and Technology Transfer, Rosenheim Technical University of Applied Sciences, Hochschulstraße 1, 83024 Rosenheim, Germany; 2 Institute for Medical Information Processing, Biometry and Epidemiology, Ludwig Maximilian University of Munich, Marchioninistraße 17, 81377 Munich, Germany; 3 Healthcare Quality Programs, Queen’s University, 84 Barrie Street, Kingston, Ontario K7L 3N6 Canada; 4 Faculty for Applied Health and Social Sciences, Rosenheim Technical University of Applied Sciences, Hochschulstraße 1, 83024 Rosenheim, Germany

**Keywords:** Systematic review, Critical pathways, Primary health care, General practitioners

## Abstract

**Background:**

Care pathways (CPWs) are complex interventions that have the potential to reduce treatment errors and optimize patient outcomes by translating evidence into local practice. To design an optimal implementation strategy, potential barriers to and facilitators of implementation must be considered.

The objective of this systematic review is to identify barriers to and facilitators of the implementation of CPWs in primary care (PC).

**Methods:**

A systematic search via Cochrane Library, CINAHL, and MEDLINE via PubMed supplemented by hand searches and citation tracing was carried out. We considered articles reporting on CPWs targeting patients at least 65 years of age in outpatient settings that were written in the English or German language and were published between 2007 and 2019. We considered (non-)randomized controlled trials, controlled before-after studies, interrupted time series studies (*main project reports*) as well as associated *process evaluation reports* of either methodology. Two independent researchers performed the study selection; the data extraction and critical appraisal were duplicated until the point of perfect agreement between the two reviewers. Due to the heterogeneity of the included studies, a narrative synthesis was performed.

**Results:**

Fourteen studies (seven main project reports and seven process evaluation reports) of the identified 8154 records in the search update were included in the synthesis. The structure and content of the interventions as well as the quality of evidence of the studies varied.

The identified barriers and facilitators were classified using the *Context and Implementation of Complex Interventions framework*. The identified barriers were inadequate staffing, insufficient education, lack of financial compensation, low motivation and lack of time. Adequate skills and knowledge through training activities for health professionals, good multi-disciplinary communication and individual tailored interventions were identified as facilitators.

**Conclusions:**

In the implementation of CPWs in PC, a multitude of barriers and facilitators must be considered, and most of them can be modified through the careful design of intervention and implementation strategies. Furthermore, process evaluations must become a standard component of implementing CPWs to enable other projects to build upon previous experience.

**Trial registration:**

PROSPERO 2018 CRD42018087689.

## Background

A care pathway or clinical pathway (CPW) is an evidence-based structured multi-disciplinary care plan that describes all relevant diagnostic and therapeutic steps in the care of patients with a specific health problem in chronological order. A CPW is used to translate evidence into local practice by considering regional conditions and demands [1, 2] as the final step of implementing evidence-based knowledge into practice. Due to the standardization of care, a CPW has the potential to reduce treatment errors, impact patient outcomes and quality of care and increase the effectiveness of health care systems [1, 3]. CPWs have been implemented in international practice since the 1980s [4] and are increasingly being used worldwide, especially in inpatient care in Australia, the USA, Canada, Europe and Asia [5], for example, with the HEART Pathway [6], the Liverpool CPW for patients with cancer [7] or CPWs for total knee arthroplasty in surgery [8]. Due to the epidemiological and demographic changes in the Western world, primary health care systems must change, and it is important to align quality of care and evidence-based practice with economic aspects and patients’ expectations. CPWs might be an answer to addressing unwanted variation in primary care (PC) that hampers reliable, patient-centred evidence-based care [9, 10]. However, there is still low utilization of CPWs in PC, even though general practitioners (GPs) see them as highly relevant [11]. Based on the important influence of contextual factors on the effectiveness of complex interventions [12] there is a low transferability of CPWs across different countries and settings when not understood adequately and reported in and adequate manner. The same applies to implementation strategies which have to be tailored and adapted to the different demands and contexts, e.g. of outpatient and inpatient care settings [2].

To develop successful implementation strategies for CPWs in PC, information about potential barriers and facilitators should be taken into account. Thus, our review addresses the following review question: Which barriers and facilitators to implementing multi-disciplinary CPWs for people aged ≥65 years in PC have been reported in the literature?

Since aged people often suffer from multimorbidity and therefore have special demands, we decided to focus on this particularly vulnerable group in PC. Vertigo, dizziness and balance disorders as frequent complaints of older people [13–16], for example, are a common reasons for their consultation in general practice [17]. Due to multifactorial etiology [18–21], the overutilization of health care in affected patients insufficiently treated in PC has been shown [22, 23].

## Methods

### Search strategy

A systematic search of literature was carried out in three electronic databases, Cochrane Library, CINAHL, and MEDLINE via PubMed. Additional sources were identified via hand searches, citation tracing and internet searches for grey literature. The initial search took place in December 19th, 2017, and a search update was conducted in July 15th, 2019. The search strategy was based on the Medline search strategy used for a Cochrane review titled *Clinical pathways for primary care: effects on professional practice, patient outcomes, and costs* [2]*,* which is currently available as protocol.

An overview of all search strategies used, terms, filters and number of results can be accessed in Additional file 1.

The review protocol was registered at PROSPERO 2018 CRD42018087689 and is available from https://www.crd.york.ac.uk/prospero/display_record.php?%20ID=CRD42018087689.

Reporting of this systematic review followed the PRISMA checklist [24].

### Selection criteria

To identify publications with relevant interventions, we used criteria as the intervention must be a structured and stepwise detailed multi-disciplinary plan that must be applied to translate evidence into practice in the local context and aims the standardization of care for a specific health problem in a specific group of patients [2]. We did not include screening, detection, risk prediction or primary preventive CPWs or pharmacological guidelines. This also refers to CPWs that deal exclusively with diagnostics and are not an intervention according to our underlying definition [2]. The target population was people aged ≥65 years in PC setting, which was defined as “[ …] products or services designed to address acute and episodic health conditions and to manage chronic health conditions. It is also [ …] where patients receive first contact care and where those in need of more specialized services are connected with other parts of the healthcare system.” [25]. Thus, we considered providers as all health professionals (HPs), including doctors as GPs and medical specialists, nurses, physical therapists, pharmacists, occupational therapists, social workers, dietitians, psychologists, and dentists involved in CPW utilization in PC setting. As patients sometimes inappropriate tend to go to the emergency rather than to their GP for reasons as intricate appointment systems and appointment availability in general practice [26], hospital stays less than 24 h were also included.

For more detail of selection criteria based on PICO construct, see Table 1.

**Table 1:** Selection criteria

Domain	Selection criteria	
Participants	People aged ≥65 years *(Operationalization according to the reported mean age of the study population of at least 60.0 years or 80% of the population aged over 60 years)*	
Setting	Primary care setting - outpatient hospital care - hospital stays < 24 h - transition from primary care to other settings Providers all health professionals including doctors as general practitioners and medical specialists, nurses, physical therapists, pharmacists, occupational therapists, social workers, dietitians, psychologists, and dentists involved in CPW utilization in PC setting	
Intervention	Criteria for considering an intervention as care pathway - (1) the intervention must be a structured, multi-disciplinary care plan that - (2) details the steps in the course of a treatment in the plan, algorithm, pathway, guide or the like and - (3) must be applied to translate evidence into practice in the local context - Aim: standardization of care for a specific health problem in a specific group of patients	
Comparator(s)	No restrictions	
Study designs	*Main project reports* - randomized controlled trials - non-randomized controlled trials - controlled before-after studies - interrupted time series	*Additional process evaluation reports* No restrictions
Outcome	No restrictions	
Publication period	2007 to 2019	
Language	- German - English	

### Study designs considered for inclusion

We included randomized controlled trials (RCTs), non-randomized controlled trials (NRCTs), controlled before-after studies (CBAs) and interrupted time series (ITS) studies, according to the Effective Practice and Organisation of Care (EPOC) study design criteria [27], written in German or English language and published from 2007 to 2019, whereby preliminary results or pilot/feasibility studies were excluded. For further detail, see Table 1.

In general, we did not exclude studies with a high risk of bias (RoB), indicating lower quality, but we did consider the RoB in the rating.

The titles, abstracts and subsequent full texts of the identified studies were screened and assessed for eligibility independently by two researchers (ES, VR). Disagreement between them was resolved through discussion, and a third reviewer (MM) was consulted if necessary. The study selection process, including deduplication, was documented, made consistent between the researchers and managed by using the Cochrane technology platform Covidence.

Since we assumed that it is possible, that barriers to and facilitators of implementation are not reported within the main publication of the respective project (*main project report*) but in independent publications, we carried out citation tracing of eligible articles to identify and include associated *process evaluation reports*.

### Data extraction and analysis

After the exclusion of non-eligible articles through the removal of obviously irrelevant reports based on the title and abstract screening and through the examination of the retrieved full texts of the potentially relevant reports, the remaining studies were extracted by using a previously piloted template based on the *EPOC good practice data extraction form* [28] supplemented by items from the *data extraction tool of the Context and Implementation of Complex Interventions (CICI) framework* [12]. If there were more relevant articles published for one original project, the various related records were extracted in one form. Data extraction forms are available from the authors on request.

The data collection process was performed by two independent researchers: ES extracted the data from all studies, and this process was duplicated by VR until the point of perfect agreement between the two reviewers. Discrepancies in the comparison of the forms were resolved by discussion and consensus.

Due to the large diversity of study characteristics and heterogeneous interventions and outcomes, a meta-analysis was not possible. Thus, a narrative synthesis following the *guidance for undertaking reviews in health care from the Centre for Reviews and Dissemination (CRD)* [29], as well as a synthesis in tabular form (see Tables 2 and 3) was undertaken.

**Table 2:** Summary of the characteristics and results of the included studies

Source, year	Design/method of data collection	Primary aim	Setting, country	Included participants: n (intervention group (IG)/ control group (CG))	Age in years: mean (range)	Groups in the intervention, provider	Results of the main project reports	Source of the barrier and facilitator data extraction
Azad et al., 2008 [30]	RCT	Effectiveness of the intervention	Primary care, Canada	Female patients with heart failure and their family caregivers 91 (45/46)	**IG:** 74.2 **CG:** 75.7 Caregivers: n.a.	**IG:** multi-disciplinary care pathway for heart failure - 12 visits - assessment and evidence-based treatment by various disciplines - group sessions/workshops: heart failure management and education **CG:** usual care **Provider:** medicine, pharmacy, nursing, occupational therapy, physical therapy, social work	No significant difference in primary outcome	
Byszewski et al., 2010 [31]	with an additional publication focusing the intervention arm	Variance, adherence		All patients from the IG (*n* = 45)	**IG:** 74.2			Barrier and facilitator typology derived from data
Bleijenberg et al., 2016a [32]	cRCT	Effectiveness of the intervention	Primary care, Netherlands	Community-dwelling elderly people 3092 (790_IGa_/ 1446_IGb_/856_CG_)	**IGa:** 73.5 **IGb:** 74.0 **CG:** 74.6	**IGa:** frailty screening followed by routine care from a general practitioner **IGb:** Proactive Primary Care Program on Preserving Daily Functioning of Older People: frailty assessment followed by personalized nurse-led care - geriatric assessment - tailored care planning - care coordination - follow-up - educational training for providers **CG:** usual care **Provider:** practice nurses, general practitioners	Significant differences in primary outcome in both IGs	
Bleijenberg et al., 2013b [33]	with a nested mixed-methods study: - quantitative: pre-and post- questionnaires - qualitative: focus groups with health professionals	Barriers, needs, expectations		32 general practitioners 21 practice nurses	General practitioners: 55.0 Practice nurses: 46.5			Barrier and facilitator themes Respondents’ agreement with pre-defined barrier statements
Bleijenberg et al., 2015 [34]	with a nested qualitative study: interviews with patients	Perceptions, experiences		11 patients from IG_b_ (subsample)	79			Barrier and facilitator themes
Bleijenberg et al., 2016b [35]	with a nested mixed-methods study: - quantitative: descriptive data - qualitative: focus group with nurses	Intervention delivery		835 patients (identified as frail) from IG_b_ Subsample of practice nurses from IG_b_ (*n* = n.a.)	Patients: 75.4 Practice nurses: n.a.			Barrier and facilitator themes
Harris et al., 2015 [36]	cRCT	Effectiveness of the intervention	Primary care, UK	Community-dwelling aged people 298 (150/148)	(60–75)	**IG:** Pedometer accelerometer consultation evaluation (PACE)-Lift intervention - individually tailored consultations - patient handbook - individual physical walking/activity plan - physical activity diary - pedometer, accelerometer - educational training for providers **CG:** usual care **Provider:** practice nurse	Positive effect on primary outcome *(significant differences at 3 months but not at 12 months),* with no effect on adverse events	
	with nested qualitative studies: interviews with patients, group interview with health professionals	Acceptability, Barriers, facilitators		30 patients 4 practice nurses				Barrier and facilitator themes
Melis et al., 2008 [37]	cRCT	Effectiveness of the intervention	Primary care, Netherlands	Community-dwelling independently living elderly people and their family caregivers 151 (85/66)	**IG:** 81.7 **CG:** 82.8 Caregivers: n.a.	**IG:** Dutch Geriatric Intervention Program (DGIP) - multi-professional assessment - individualized, integrated treatment plan - regular evaluation and follow-up visits **CG:** regular medical care **Provider:** primary care physician, geriatric specialist nurse (and geriatricians)	The intervention had a positive effect on primary outcomes *(significant differences at 3 months but not at 6 months)*	
Melis et al., 2010 [38]	with a nested process evaluation	Content, adherence		All patients from the IG (*n* = 85)	81.7			Barrier and facilitator typology derived from data
Metzelthin et al., 2013b [39]	cRCT	Effectiveness of the intervention	Primary care, Netherlands	Community-dwelling frail elderly people 346 (193/153)	**IG:** 77.5 **CG:** 76.8	**IG:** Prevention of Care (PoC) approach- multi-dimensional assessment - interdisciplinary care - tailored treatment plan - evaluation and follow-up - educational training for providers **CG:** usual care **Provider:** practice nurses, general practitioners, occupational therapists, physical therapists	No significant differences in primary outcomes	
Metzelthin et al., 2013a [40]	with additional mixed-method components: - quantitative: logbooks, evaluation forms - qualitative: interviews with patients and health professionals, focus groups with health professionals	Extent to which the implementation occurred as planned, experiences regarding benefits, burden, barriers and facilitators		7 practice nurses 12 general practitioners 6 occupational therapists 20 physical therapists 194 patients	Patients: 77.7 Health professionals: n.a.			Barrier and facilitator themes
van Bruggen et al., 2008 [41]	cRCT	Effectiveness of the intervention	Primary care, Netherlands	People with type 2 diabetes 1640 (822/818)	**IG:** 67.1 **CG:** 67.2	**IG:** - locally adapted shared care guidelines - educational training for providers **CG:** usual care (national guidelines) **Provider:** general practitioners, nurses, practice assistance	No significant differences in outcomes, but improvement in the process of diabetes care	
	with nested qualitative studies: interviews with health professionals	Barriers, facilitators						Barrier and facilitator themes
Weldam et al., 2017a [42]	cRCT	Effectiveness of the intervention	Primary care, Netherlands	People with mild to severe COPD 204 (103/101)	**IG:** 68.0 **CG:** 66.0	**IG:** nurse-led Chronic Obstructive Pulmonary Disease – Guidance, Research on Illness Perception (COPD-GRIP) intervention - three extra face-to-face consultations with individualized content, based on the patient’s responses and the needs - assessment - individualized care plan - evaluation - educational training for providers **CG:** usual care **Provider:** practice/respiratory nurse	No significant difference in outcomes	
Weldam et al., 2017b [43]	with nested mixed-method components: - quantitative: pre- and post- questionnaires - qualitative: focus groups with health professionals	Facilitators, barriers, expectations		24 nurses	Questionnaires: 45.5 Focus group: 47.4			Barrier and facilitator themes Respondents’ agreement with pre-defined barrier statements

*IG* intervention group; *CG* control group; *COPD-GRIP* Chronic Obstructive Pulmonary Disease – Guidance, Research on Illness Perception; *DGIP* Dutch Geriatric Intervention Program; *PACE* Pedometer accelerometer consultationevaluation; *PoC* Prevention of Care

**Table 3:** Overview of the reported barriers and facilitators

Domain*	Barriers	Facilitators
**CONTEXT**		
** Geographical context**	–	–
** Epidemiological context**	Multi-morbidity [31, 33, 43] People aged ≥85 years [33] Mental health problems [35]	–
** Socio-cultural context**	Cultural background [33, 43] Low health literacy [43] Gender [33, 43] Frequency of general practice visits [33, 43]	–
** Socio-economic context**	Low socio-economic status [33, 43]	–
** Ethical context**	–	–
** Legal context**	–	–
** Political context**	Lack of financial incentives/compensation [33, 41, 43]	–
**IMPLEMENTATION**		
** Implementation theory**	–	–
** Implementation process**	–	–
** Implementation strategies**	Overload of information in training activities for health professionals [40]	Training and educational activities for health professionals [33, 36, 41] Handbook as a clear guideline for health professionals [43]
** Implementation agents**		
*Health professionals*		
*Knowledge and skills*	Insufficient knowledge [33, 40, 41] Lack of competence [40] Lack of experience [40]	Professional skills [33, 40, 43] Organizational skills [40] Communication skills [40] Empathic capacity [40]
*Behaviour-related factors*	Lack of motivation [41] Initial difficulties in implementation due to changes in routines [40, 43] Negative attitudes towards intervention [33] Reluctance regarding an intervention component [41, 43]	Positive expectations regarding intervention [33, 43] Type of recommendation [38]
*Interaction-related factors*	Communication and collaboration issues [33] Difficulties in organizing team meetings [40] Insufficient involvement of professionals [33]	Interdisciplinary communication and cooperation [33, 35, 40] Intradisciplinary communication and cooperation [33, 41] Sufficient involvement of family caregivers [34] Clear responsibilities [33, 40]
*Application of the intervention*	Time expenditure [33, 40, 43] Complexity of intervention [33, 40]	Individual, flexible, tailored intervention [33, 43] Practicable layout [43] Good fit of the intervention to daily practice [43]
*Patients*		
*External assessment*		
*Behaviour-related factors*	Low treatment adherence [33, 38, 43]	–
* External factors influencing* * adherence*	Transportation issues [31] Scheduling problems [31]	–
* Self-assessment*		
*Behaviour-related factors*	–	Positive expectations regarding intervention [33, 40]
*Components of intervention*	High temporal expenditure effort [40] High bureaucratic effort [36] Difficulties in distinguishing the involved disciplines [40]	Interventions tailored to individual needs [33, 34, 36] Possibility for adaptation [40] Close monitoring of changing situations [34] Provision of written advice [36] Use of technical devices for outcome measurement [36]
*Interaction with health professionals*	–	Personal meetings with health professionals [36, 40] Good professional-patient relationship [33, 34, 40] Good internal exchange between HPs [34]
** Implementation outcomes**	Difficulties in identifying the appropriate target group [33, 40]	–
**SETTING**		
*Work environment*	Lack of available staff [31, 33] Lack of sufficiently educated staff [33] Lack of time [33, 35, 41, 43] Lack of space [31, 43] Discontinuity [34]	Transparency about referral possibilities [33]

*CICI framework domains are **bolded**, additional categories are in *italics*

### Critical appraisal

The critical appraisal was carried out by two independent researchers (the critical appraisal was conducted in its entirety by ES and then duplicated by VR until the point of perfect agreement between the two reviewers), and a third reviewer (MM) was involved if necessary.

We used the *Cochrane Collaboration’s tool for assessing RoB* for (N)RCTs and CBAs by completing the RoB table via Review Manager (RevMan) 5.3 software [44]; in cluster randomized trials, we also considered the risk of particular bias as recommended by the *Cochrane Handbook for Systematic Reviews of Interventions* [45]; in ITS we used the seven standard criteria [46]. We judged each domain as being at *low*, *high*, or *unclear risk* (Additional file 2) and created a RoB summary figure (see Additional file 3) and a graph to illustrate the proportion of studies with each of the judgements (see Fig. 2).

For the process evaluation reports, we used the *Critical Appraisal Skills Programme* (CASP) *Checklist* for qualitative research [47] and the *Mixed Methods Appraisal Tool* (MMAT) [48]. An overview of critical appraisal tools used for the included study designs is given in Additional file 4.

## Results

### Study selection

**Fig. 1:**
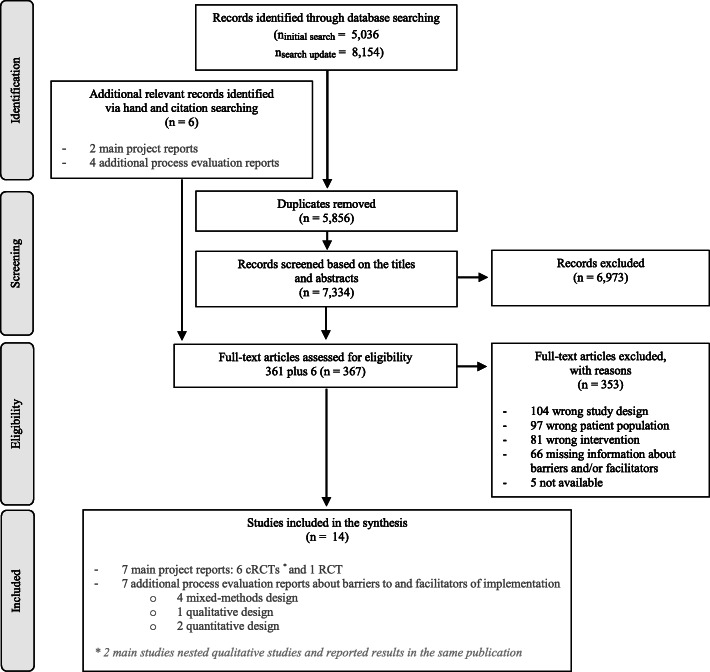
PRISMA flow chart

The search generated 8154 hits. After removing duplicates and irrelevant publications based on the title and abstract screening, we assessed 367 full-text articles for eligibility, six of which originated from the additional hand and citation searching. After the exclusion of 353 articles (see Fig. 1 for the PRISMA flow chart), a total of 14 studies (seven main project reports and seven process evaluation reports) were included in the synthesis.

The presentation of the results is based on the different included CPWs of the seven main project reports.

### Characteristics of included studies

One main project report was a RCT [30] and six were cluster RCTs (cRCTs) [32, 36, 37, 39, 41, 42]. Two included nested process evaluation components in the main report [36, 41] and for five additional process evaluation reports were published separately. Details on the characteristics and results of the included studies can be found in Table 2.

The studies were published between 2008 and 2017 and took place in PC settings in three different countries: five in the Netherlands [32, 37, 39, 41, 42], one in the UK [36] and one in Canada [30].

The included projects comprised 5822 participants (3634 patients in intervention groups; 2188 patients in control groups).

The mean ages in the intervention groups ranged from 67.1 to 81.7 years and from 66.0 to 82.8 years in the control groups. One study only reported overall age range and did not report mean age [36].

All projects compared CPWs with usual care to assess their effectiveness. Three projects tested a CPW for persons with specific health conditions, which were type 2 diabetes [41], chronic obstructive pulmonary disease (COPD) [42], and heart failure [30]. The other projects targeted on community-dwelling people [32, 36, 37, 39]. More detailed information about the study characteristics and the results of single studies can be found in Table 2.

Despite the general diversity of the seven CPWs, there were commonalities with regard to the development and structure of the interventions. Thus, e.g. the development of all interventions was evidence-based, and four studies reported the involvement of clinicians. A total of six CPWs provided an individually tailored treatment. Education and training for health care providers was included in six CPWs. More detailed information about the structure of the interventions is displayed in Additional file 5. No project provided a clear and comprehensive distinction between intervention components and used implementation strategy. For details of the components of the seven CPWs, see Table 2.

Detailed information about characteristics of excluded studies and reasons for exclusion are available from the authors upon reasonable request.

### Outcome measures

Five projects used patient-relevant primary outcomes, such as disability [39], daily functioning [32], functional performance in activities of daily living and mental well-being [37], quality of life and functional capacity for older females living with heart failure [30] and health status of COPD patients [42]. Two studies investigated surrogate endpoints, such as changes in average daily step count [36] and the percentage of people with poor glycaemic control [41].

### Quality of evidence

**Fig. 2:**
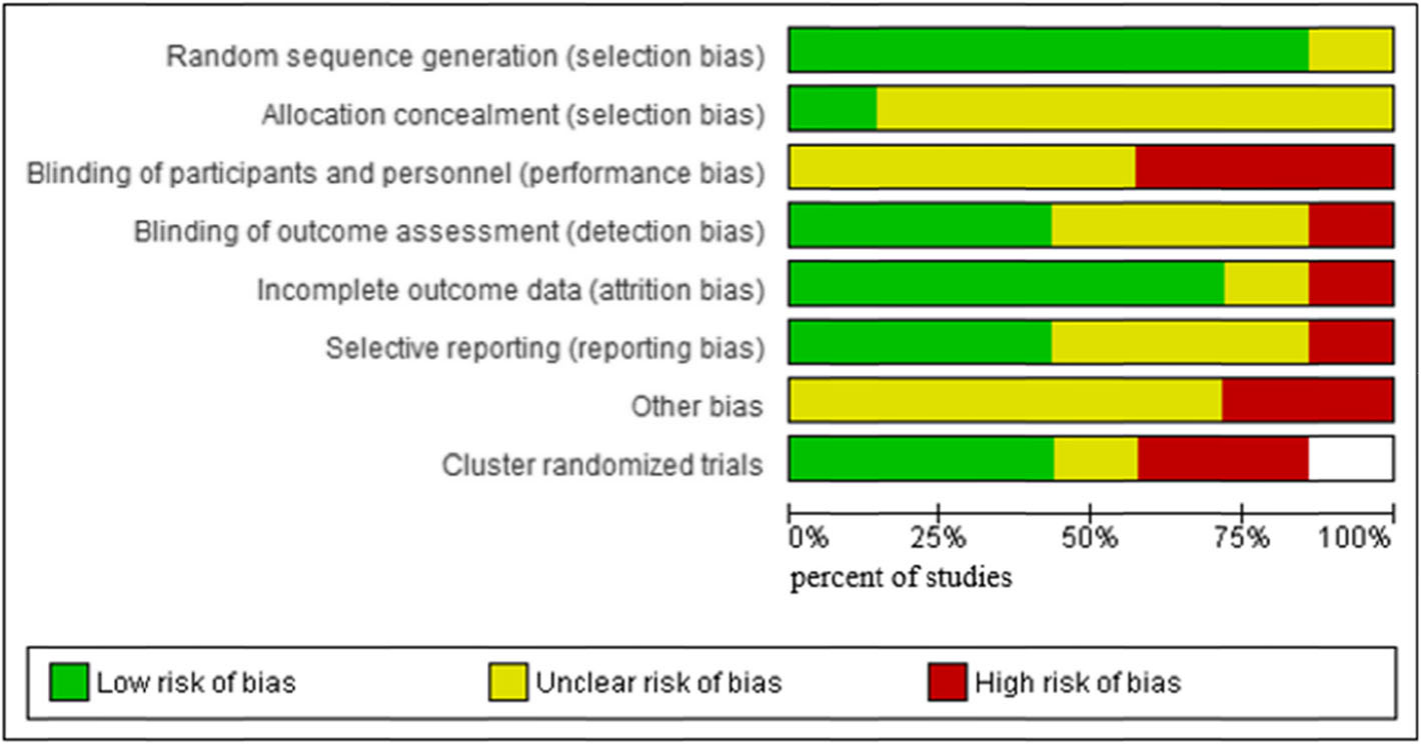
Risk of bias graph of RCTs and cRCTs (designed by using RevMan [44])

Details of the judgements about each RoB item in the included (cluster-)randomized controlled studies and across these trials are shown in Additional file 2, Additional file 3 and Fig. 2.

Due to a lack of information in almost all studies, the authors judged a total of 43,6% (*n* = 24/55) of RoB domains as being *unclear* (38,2% as *low risk*: *n* = 21/55; 18,2% as *high risk*: *n* = 10/55). For a detailed information on RoB assessment see Fig. 2 and Additional file 3.

The problem of poor reporting was also relevant in the quality assessment of the process evaluation reports (see Additional file 6 for CASP and Additional file 7 for MMAT). None of the studies that use qualitative methods adequately described the relationship and interaction between the participants and the researcher. This also applies to qualitative parts of mixed-methods studies. One qualitative study did not report approval of an ethics committee or institutional review board.

### Factors influencing the success of implementation

The classification of barriers to and facilitators of successful implementation of CPWs in PC was based on the context, implementation and setting dimensions of the *CICI framework* [12].

An overview of barriers and facilitators in the individual studies is shown Table 3. Barriers were most frequently identified within the dimensions of implementation agents (*n* = 7) and setting (*n* = 4). Facilitators were most frequently determined within the implementation agents (*n* = 6) and implementation strategies (*n* = 4) (see Table 4).

**Table 4:** Distribution of barriers and facilitators

Source of main project report, year	Barriers													Facilitators												
	Context							Implementation					Setting	Context							Implementation					Setting
	Geographical context	Epidemiological context	Socio-cultural context	Socio-economic context	Ethical context	Legal context	Political context	Implementation theory	Implementation process	Implementation strategies	Implementation agents	Implementation outcomes		Geographical context	Epidemiological context	Socio-cultural context	Socio-economic context	Ethical context	Legal context	Political context	Implementation theory	Implementation process	Implementation strategies	Implementation agents	Implementation outcomes	
Azad et al., 2008 [30]		X									X		X													
Bleijenberg et al., 2016a [32]		X	X	X			X				X	X	X										X	X		X
Harris et al., 2015 [36]											X												X	X		
Melis et al., 2008 [37]											X													X		
Metzelthin et al., 2013b [39]										X	X	X												X		
van Bruggen et al. 2008 [41]							X				X		X										X	X		
Weldam et al., 2017a [42]		X	X	X			X				X		X										X	X		

### Context

Three CPWs considered aspects of the *epidemiological context* such as multi-morbid [31, 33, 43] patients aged at least 85 years [33] with mental health problems [35] as barriers to applying an intervention.

Two of the CPWs reported the cultural background [33, 43], a low health literacy [43] and gender [33, 43] as potential barriers that could be attributed to the domain of *socio-cultural context*. Such patient-related characteristics can lead to a time lag in the application of an intervention. Additionally, the frequency of general practice visits [33, 43] have been reported to have a negative impact by two CPWs and could therefore be seen as barrier according to two CPWs.

Additionally, two CPWs considered a low socio-economic status [33, 43] within the domain of *socio-economic context* as barriers to applying an intervention.

Furthermore, aspects related to the *political context*, such as a lack of an incentive systems [41] or adequate reimbursement models [43] or absent monetary compensations [33], were reported in three CPWs as potential barriers for the effective implementation of an intervention.

No barriers or facilitators within the domains *geographical, ethical* and *legal context* could be identified. None of the CPWs described facilitators in any of the dimensions of the domain context.

### Implementation

Within the domain of *implementation strategies* the involved HPs of three CPWs emphasized the importance of training activities and reported appropriate training and education in applying an intervention [33, 36, 41] as facilitator. One CPW considered an overload of information during training activities as potential barrier [40]. According to the results of one CPW, a handbook as facilitator can serve as a clear guideline for HPs to promote a structured application of intervention [43].

The domain of *implementation agents* can be divided into the two areas of HPs and patients.

On the one hand, HPs’ insufficient or even lack of knowledge about how to perform intervention components such as assessments or tests [33, 40, 41], their lack of competence in general [40] and their insufficient experience and job training [40] were considered barriers regarding knowledge and skills in three CPWs. On the other hand, three CPWs identified knowledge and skills such as professional [33, 40, 43], organizational [33] and communication skills [33] and empathic capacity [33] as serving as facilitators to the implementation of the approach. The behaviour-related factors of attitude and awareness, such as a lack of motivation of end-users [41] (*n* = 1) and initial difficulties in implementation due to changes in routines [40, 43] (*n* = 2) were reported as barrieres, which can reduce the success of intervention. Further barriers were negative attitude towards the intervention, such as doubts about the expected results [33] in one CPWs, and reluctance regarding an intervention component due to a lack of agreement [41, 43] in two included CPWs, e.g., the prescription of multiple drug regimes [41]. In contrast, a positive attitude towards the effectiveness of the intervention [33, 43] is reported to be a facilitator according to two CPWs. One CPW stated that interventions that provide recommendations to both patients and GPs increased adherence among HPs and affected patients and are therefore facilitators [38].

Interaction-related factors were identified in five CPWs as influencing aspects. In this regard, HPs named communication and collaboration issues [33] and difficulties in organizing team meetings [40] as barriers. HPs considered good interdisciplinary communication and cooperation [33, 35, 40] in two included CPWs as well as clear roles and task definition [33, 40] in two CPWs as facilitators. In addition to the consideration of the multi-disciplinary team, the positive impact of intradisciplinary communication and cooperation was identified in two included CPWs as a facilitator [33, 41], e.g., by making comparisons with peers [41]. The integration of family caregivers into the intervention, if possible, was identified as facilitator in one CPW [34], whereas insufficient involvement of single professions was mentioned as barrier in one CPW [33]. According to three CPWs, further barriers in application of the CPW arise due to the extent of intervention, such as time-consuming parts [33, 40, 43] and overly complex intervention components [33, 40]. Two CPWs reported an individual, flexible, tailored intervention customized to patients’ needs, wishes and preferences providing the HPs as major facilitator in application [33, 43]. Another facilitator in implementation is a good fit of the intervention to the day-to-day work of the delivery agents [43]. A practicable layout of the intervention can ease adoption in daily practice [43] as facilitator sccording to one included CPW.

In addition to HPs, patients as consumers of the intervention, were also considered to affect implementation success. Aspects in this domain were partly identified by the patients themselves (self-assessments) and partly by HPs based on their experiences with affected patients (external assessments): regarding behaviour-related factors, HPs in three CPWs assumed patients’ motivational issues to be a reason for their low treatment adherence and therefore as barrier [33, 38, 43]. Furthermore, external factors such as transportation issues, sometimes due to adverse weather conditions or scheduling conflicts with other appointments, affected the adherence of intervention recipients and serve as barriers [31]. Similar to HPs, patients in two studies also indicated that positive expectations regarding interventions [33, 40] were a facilitator. The delivery was also affected by the structure of the intervention components. Participants of one CPW perceived high temporal expenditure due to time-consuming participation to be a barrier [40]. Recipients of each one CPW classified high bureaucratic effort [36] and difficulties in distinguishing the involved disciplines [40] as barriers. On the other hand, two CPWs reported tailored interventions meeting patients’ current needs [33, 34, 36]; one CPW the possibility for adaptations to avoid excessively restricting their own decision making, e.g., through self-management approaches [40]; and one CPW close monitoring of changing situations, which transmits a sense of security [34], as facilitators. Furthermore, in one CPW the provision of written advice such as a handbook [36] and the use of technical devices for outcome measurement [36] were seen as facilitators by consumers. In addition, patients considered interactions with HPs through personal meetings [36, 40] in two CPWs, good professional-patient relationships [33, 34, 40] in two CPWs and good internal exchange between HPs [34] in one CPW to be facilitators.

Within the domain of *implementation outcomes* two CPWs reported a barrier in problems occurred during the identification of the appropriate target group as the first step of the intervention [33, 40], e.g., due to dysfunctional screening methods [40].

No barriers or facilitators within the domains *implementation theory* and *implementation process* were reported. In addition, no facilitators within the domain of *implementation outcomes* were mentioned by included CPWs.

### Setting

Barriers reported in four CPWs within the *work environment* in the dimension of setting are inadequate staffing due to the general lack of available staff [31, 33], e.g., due to illness or part-time employment [31] and lack of sufficiently educated staff [33]. Structural conditions lead to time pressure [33, 35, 41, 43], e.g., due to excessive workload in daily practice [35, 43], which negatively affects the situational performance of intervention components. Additionally, two CPWs mentioned a lack of space as barrier [31, 43]. Also, one CPW cited discontinuity problems in GPs as a barrier [34]. Transparency about referral possibilities promoting the familiarity of HPs with these options was identified as a facilitator [33].

## Discussion

This study analysed barriers to and facilitators of the implementation of CPWs in PC to gain a better understanding of the factors needed for their successful implementation.

We found that the implementation of interventions into practice requires changes and adaptations in the knowledge, attitudes and behaviour of HPs to achieve a positive impact on outcomes. The finding on the negative influence of personal factors of HPs, such as their lack of knowledge and their attitudes, is in line with findings from a review about barriers and strategies in guideline implementation [49] and a review of staff-reported barriers and facilitators to implementation of hospital-based, patient-focused interventions [50]. Our results show that appropriate training activities for HPs are particularly relevant, as confirmed by a larger feasibility study evaluating a local coronary heart disease treatment pathway in PC [51]. Two systematic reviews focusing on in-hospital settings showed similar results [49, 50]. We found that HPs considered the use of a structured, step-by-step explanatory handbook as a facilitator [43]. This finding is in line with the results of a feasibility study in PC [37]. Findings from another feasibility study suggested that additional material such as small portable cards with inclusion criteria, telephone numbers and listed referral options are helpful [52]. A meta-analysis of the effectiveness of implementation strategies for non-communicable disease guidelines in primary health care concluded that the simple provision of educational materials without training is ineffective [53]. In line with our findings, a review on secondary care found that providing information about successful examples can lower implementation barriers and enhance adherence [50]. Regarding the results showing that HPs have difficulties accepting interventions due to negative attitudes or reluctance regarding intervention components, similar studies also stated that it seems to be advisable to integrate local end-users into the development and implementation process [49, 51], which is in line with the *UK Medical Research Council* (MRC) *guidance* that recommends involving local end-users to promote successful long-term establishment of effective intervention in practice [54].

Our results show that intervention success also depends on patients’ acceptance and adherence, e.g., due to the risk of a lack of understanding of recommendations. The identified facilitators such as precise and thoroughly explained recommendations [38] as well as the provision of written advice for patients [36] seem to be easy to use in practice. Reasons for negative attitudes towards interventions must be analysed individually to find solutions to promote acceptance and adherence. We also found that the application of an intervention can be made more difficult and time consuming due to several unavoidable patient-related factors, such as age [33], multi-morbidity [31, 33, 43] and cultural background [33, 43]. To counteract this difficulty, CPWs should be designed to be truly contextualised to the local settings, as well as taking into consideration common issues faced by the elderly age group.

We identified a good fit of the intervention with the day-to-day work of the delivery agents as a facilitator [43]. To promote a good fit, other studies suggested the integration of interventions into practice software in PC [51] or the use of tablets or smartphones in in-hospital settings [49]. Metzelthin et al. [40], in relation to a process evaluation of the implementation of a nurse-led care approach for community-dwelling frail older people, observed that digitalization of forms may additionally favour interdisciplinary exchange of data. Our results showed that clearly defined responsibilities with regard to tasks and roles are the basic prerequisite for multi-disciplinary communication and cooperation to promote efficient healthcare delivery [33, 40], which is in line with findings for in-hospital settings [49]. These findings underline the importance of the careful CPWs design in order to build upon current practice and take into account day-to-day practice to ensure the uptake by HPs. Since we identified a lack of time [33, 35, 41, 43] as well as overly time-consuming [33, 40, 43] and complex [33, 40] intervention components as barriers, the CPW application should not be associated with too much effort, especially since HPs are already under time pressure. Recommendations and tools have to be plausible, clear and transparent and be presented in a user-friendly, simplified and short form, consistent with findings for in-hospital settings [49, 50]. Furthermore, they must be evidence-based, which is in line with findings in PC [51] as well as with secondary care setting [49]. Thus, Kramer et al. [51] stated that recommendations must conform to the advice of guidelines or other (inter)national guidance to avoid contradictory or overlapping recommendations, whereas an integration into a larger geographic context may facilitate implementation.

A lack of financial incentives and compensation [33, 41, 43] were reported to be important barriers. To overcome this issue, projects should plan to use case payments, and new reimbursement options should be considered to facilitate long-term implementation.

Notably, the retrieved studies originated from a few different studies, and most of them were conducted in the Netherlands [32, 37, 39, 41, 42].

### Limitations

This systematic review has some limitations. An important issue is the evaluation of the main inclusion criterion. The terms *care pathways* and *critical pathways* were not consistently used in the literature. We tried to overcome this issue by applying a broad definition of CPWs [2] to allow for consistency among the compared studies. Eventhough both the European Pathway Association (E-P-A) in 2007 [55] and a Cochrane review from 2010 [3] indicated that CPWs have to be considered as a complex intervention, it seems not to be common sense [54] that therefore, CPWs have to be developed and evaluated in a specific manner. This might explain the lack of systematic and rigorous investigation of the context, in terms of barriers and facilitators that would allow thorough evaluation of the external validity of the implemented CPWs.

### Transferability of review results

Despite the general interest of GPs in CPWs, there is a low utilization of CPWs in PC [11]. Therefore, the included studies in this systematic review were conducted in the UK, Canada, and the Netherlands. This limits the transferability of our findings to similar healthcare contexts. It is obvious that the transferability of our findings might be limited to similar healthcare contexts with a strong gate-keeper role of the GP in PC, and the publicly funded healthcare systems in the UK and Canada [56]. The Dutch healthcare system is based on a different funding model, but with the same gatekeeper role of GPs to refer patients to specialists which are based at hospitals.

The varying funding mechanisms in the different countries were the primary studies were conducted may represent another limitation. The publicly funded (tax-based) healthcare systems in the UK and Canada differ significantly from the Dutch system. The Dutch system is funded by a dual system that came into effect in January 2006 [56]. It consists of a publicly funded component, and via a basic healthcare insurance package which is mandatory. Every Dutch resident has to choose their basic insurance package in order to define the scope of the healthcare services provided [56]. This means that the transferability of our systematic review findings are limited to countries with a similar healthcare system. Moreover, the financial incentives offered in the Dutch healthcare system could be confounding mechanisms or facilitators of successful implementation itself, and not the CPW as a causal factor [56].

In addition, the poor quality of reporting in terms of missing information for many core items made a straightforward assessment of internal validity difficult and might have led to inappropriate downgrading. We are, however, confident that our rigorously applied approach and reporting of all steps makes the conclusions transparent.

## Conclusions

In the implementation of CPWs in PC practice, a multitude of barriers and facilitators must be considered, and most of them can be modified through careful design of intervention and implementation strategies. We observed a lack of transparent and comprehensive reporting of the intervention components, their implementation strategies and contexts. There is an urgent need to improve the quality of research on CPWs and to follow the established guidelines in conducting and reporting research involving comprehensive process evaluations to produce reliable and transferable evidence to make this promising technology available for practice.

## Supplementary information

**Additional file 1.** Overview of literature database search strategies, used search terms, filters and number of results.

**Additional file 2.** Methodological quality of included main project reports.

**Additional file 3.** Risk of bias summary of RCTs and cRCTs (designed by using RevMan [44]).

**Additional file 4.** Overview of critical appraisal tools used for different study designs.

**Additional file 5.** Main components of the interventions reported in the included main project reports.

**Additional file 6.** Quality assessment results of aspects of the qualitative studies (CASP Checklist).

**Additional file 7.** Quality assessment results of aspects of the mixed-method studies (MMAT).

## Data Availability

Data extraction forms are available from the authors on request.

## Abbreviations

CASP: Critical Appraisal Skills Programme
CBA: Controlled before-after study
CG: Control group
CICI: Context and Implementation of Complex Interventions
COPD: Chronic obstructive pulmonary disease
CPW: Care pathway
cRCT: Cluster randomized controlled trial
CRD: Centre for Reviews and Dissemination
E-P-A: European Pathway Association
EPOC: Effective Practice and Organisation of Care
GP: General practitioner
HP: Health professional
IG: Intervention group
ITS: Interrupted time series
MMAT: Mixed Methods Appraisal Tool
MRC: Medical Research Council
NRCT: Non-randomized controlled trial
PC: Primary care
PICO: Population, Intervention, Comparison(s) and Outcome
RCT: Randomized controlled trial
RoB: Risk of bias
