# Supplementary material for: Barriers to and facilitators of the implementation of multi-disciplinary care pathways in primary care: a systematic review
Source: BMC Fam Pract. 2020 Jun 19;21:113. doi: 10.1186/s12875-020-01179-w (PMC7305630; doi:10.1186/s12875-020-01179-w)
Supplement: Supplementary file 1 — Additional file 1. Overview of literature database search strategies, used search terms, filters and number of results. [file 12875_2020_1179_MOESM1_ESM.docx]

**Additional file 1** Overview of literature database search strategies, used search terms, filters and number of results

| **Database** | **Date** | **Search terms** | | | | | | **Additional**  **filters** | **Results** |
| --- | --- | --- | --- | --- | --- | --- | --- | --- | --- |
|  |  | **No** | **Terms related to care pathways** | **No** | **Terms related to setting** | **No** | **Terms related to study designs** |  |  |
| **MEDLINE via**  **PubMed** | 2017/12/19 | #1 | clinical pathway?[Title] | #24 | "Primary Health Care"[Mesh] | #41 | randomized controlled trial[Publication Type] | **Publication date from 2007/01/01 to 2017/12/31;**  **English;**  **German;**  **80 and over: 80+ years; Aged: 65+ years** |  |
|  |  | #2 | "Critical Pathways"[Mesh] | #25 | "Family Practice"[Mesh] | #42 | controlled clinical trial[Publication Type] |  |  |
|  |  | #3 | (clinical[Title/Abstract] OR  critical[Title/Abstract]) AND  (pathway?[Title/Abstract] OR  path?[Title/Abstract]) | #26 | "Physicians, Primary Care"[Mesh] | #43 | multicenter study[Publication Type] |  |  |
|  |  | #4 | (care[Title/Abstract] AND  algorithm?[Title/Abstract]) OR  clinical algorithm?[Title/Abstract] | #27 | "General Practice"[Mesh] | #44 | pragmatic clinical trial[Publication Type] |  |  |
|  |  | #5 | (care[Title/Abstract] AND  pathway?[Title/Abstract]) | #28 | "Physicians, Family"[Mesh] | #45 | (randomis*[Title/Abstract] OR  randomiz*[Title/Abstract] OR  randomly[Title/Abstract]) |  |  |
|  |  | #6 | (treatment[Title/Abstract] AND  algorithm?[Title/Abstract]) | #29 | "General Practitioners"[Mesh] | #46 | groups[Title/Abstract] |  |  |
|  |  | #7 | (management protocol?[Title/Abstract]) OR treatment protocol?[Title/Abstract] | #30 | "Outpatient Clinics, Hospital"[Mesh] | #47 | (trial[Title] OR multicenter[Title] OR  multi center[Title] OR  multicentre[Title] OR  multi centre[Title]) |  |  |
|  |  | #8 | (care[Title/Abstract]) AND (plan?[Title/Abstract] OR map?[Title/Abstract]) | #31 | "Ambulatory Care"[Mesh] | #48 | intervention?[Title/Abstract] OR  effect?[Title/Abstract] OR  impact?[Title/Abstract] OR  controlled[Title/Abstract] OR  control group?[Title/Abstract] OR  (before[Title/Abstract] AND  after[Title/Abstract]) OR  (pre[Title/Abstract] AND  post[Title/Abstract]) OR  ((pretest[Title/Abstract] OR  pre test[Title/Abstract]) AND  (posttest[Title/Abstract] OR  post test[Title/Abstract])) OR  quasiexperiment*[Title/Abstract] OR  quasi experiment*[Title/Abstract] OR  pseudo experiment*[Title/Abstract] OR  pseudoexperiment*[Title/Abstract] OR  evaluat*[Title/Abstract] OR  time series[Title/Abstract] OR  time point?[Title/Abstract] OR  repeated measur*[Title/Abstract] |  |  |
|  |  | #9 | (protocol?[Title/Abstract]) AND  (nursing[Title/Abstract] OR  directed[Title/Abstract] OR  guided[Title/Abstract]) | #32 | "Ambulatory Care  Facilities"[Mesh] | #49 | "Non-Randomized Controlled  Trials as Topic"[Mesh] |  |  |
|  |  | #10 | ((local[Title/Abstract] OR  locally[Title/Abstract]) AND  adapt*[Title/Abstract]) AND  guideline?[Title/Abstract] | #33 | "Community Health  Services"[Mesh] | #50 | "Interrupted Time Series  Analysis"[Mesh] |  |  |
|  |  | #11 | (treatment model?[Title/Abstract]) AND standardi*[Title/Abstract] | #34 | "Community Health  Centers"[Mesh] | #51 | "Controlled Before-After  Studies"[Mesh] |  |  |
|  |  | #12 | (standardi*[Title/Abstract]) AND  protocol?[Title/Abstract] | #35 | (primary[Title/Abstract] OR  communit*[Title/Abstract]) AND  (care[Title/Abstract] OR  health*[Title/Abstract]) | #52 | #41 OR #42 OR #43 OR #44 OR #45 OR #46 OR #47 OR #48 OR #49 OR #50 OR #51 |  |  |
|  |  | #13 | systematic detection[Title/Abstract] | #36 | (family practi*[Title/Abstract] OR  family doctor*[Title/Abstract] OR  family physician*[Title/Abstract] OR  gp*[Title/Abstract] OR  general practi*[Title/Abstract]) |  |  |  |  |
|  |  | #14 | #2 OR #3 OR #4 OR #5 OR #6 OR #7 OR #8 OR #9 OR #10 OR #11 OR #12 OR #13 | #37 | (outpatient?[Title/Abstract] OR  ambulatory[Title/Abstract]) AND  (care[Title/Abstract] OR  healthcare[Title/Abstract] OR  clinic?[Title/Abstract] OR  service?[Title/Abstract] OR  facilit*[Title/Abstract]) |  |  |  |  |
|  |  | #15 | "Clinical Protocols"[Mesh] | #38 | #24 OR #25 OR #26 OR #27 OR #28 OR #29 OR #30 OR #31 OR #32 OR #33 OR #34 OR #35 OR #36 OR #37 |  |  |  |  |
|  |  | #16 | (treat*[Title/Abstract]) OR  therap*[Title/Abstract] |  |  |  |  |  |  |
|  |  | #17 | #15 AND #16 |  |  |  |  |  |  |
|  |  | #18 | "Practice Guidelines as Topic"[Mesh] |  |  |  |  |  |  |
|  |  | #19 | ((implement*[Title/Abstract]) OR  pathway[Title/Abstract]) OR  protocol?[Title/Abstract] |  |  |  |  |  |  |
|  |  | #20 | #18 AND #19 |  |  |  |  |  |  |
|  |  | #21 | (guideline?[Title/Abstract]) AND  (implement*[Title/Abstract] OR  pathway[Title/Abstract] OR  protocol?[Title/Abstract]) |  |  |  |  |  |  |
|  |  | #22 | #20 OR #21 |  |  |  |  |  |  |
|  |  | #23 | #14 OR #17 OR #22 |  |  |  |  |  |  |
|  |  |  | | | | | |  |  |
|  |  | #39 | #23 AND #38 | | |  |  |  |  |
|  |  | #40 | #1 OR #39 |  |  |  |  |  |  |
|  |  |  | | | | | |  |  |
|  |  | **#53** | **#40 AND #52** | | | | |  | **3,558** |
|  | 2019/07/15 | **#53** | **#40 AND #52** | | | | | **Extended publication date to 2019/12/31** | **4,580** |
| **CINAHL** | 2017/12/19 | S1 | TI clinical N2 pathway? | S24 | (MH "Primary Health Care") | S38 | PT randomized controlled trial | **Published date: 2007/01/01-2017/12/31;**  **Narrow by language: English,**  **German;**  **Narrow by subject age:**  **aged: 65+ years, aged: aged, 80 & over** |  |
|  |  | S2 | (MH "Critical Path") | S25 | (MH "Family Practice") | S39 | PT clinical trial |  |  |
|  |  | S3 | (clinical or critical) N1  (pathway? or path?) | S26 | (MH "Physicians, Family") | S40 | PT research |  |  |
|  |  | S4 | (care N2 algorithm?) or  clinical algorithm? | S27 | (MH "Ambulatory Care Facilities") | S41 | (MH "Randomized Controlled  Trials") |  |  |
|  |  | S5 | care N1 pathway? | S28 | (MH "Outpatient Service") | S42 | (MH "Clinical Trials") |  |  |
|  |  | S6 | treatment N3 algorithm? | S29 | (MH "Ambulatory Care") | S43 | (MH "Intervention Trials") |  |  |
|  |  | S7 | (management protocol?) or  (treatment protocol?) | S30 | (MH "Community Health  Services+") | S44 | (MH "Nonrandomized Trials") |  |  |
|  |  | S8 | care N1 (plan? or map?) | S31 | (MH "Community Health  Centers+") | S45 | (MH "Experimental Studies") |  |  |
|  |  | S9 | protocol? N1 (nursing or  directed or guided) | S32 | (primary or communit*) N5 (care or health*) | S46 | (MH "Pretest-Posttest Design+") |  |  |
|  |  | S10 | (local or locally) N2 adapt* N5  guideline? | S33 | family practi* or family doctor* or family physician* or gp* or  general practi* | S47 | (MH "Quasi-Experimental  Studies+") |  |  |
|  |  | S11 | (treatment model?) N10 standardi* | S34 | (outpatient? or ambulatory) N2 (care or healthcare or clinic? or service? or facilit*) | S48 | (MH "Multicenter Studies") |  |  |
|  |  | S12 | standardi* N3 protocol? | S35 | S24 OR S25 OR S26 OR S27 OR S28 OR S29 OR S30 OR S31 OR S32 OR S33 OR S34 | S49 | (MH "Health Services Research") |  |  |
|  |  | S13 | systematic detection |  |  | S50 | TI (randomis* or randomiz* or randomly) OR AB (randomis* or randomiz* or  randomly) |  |  |
|  |  | S14 | S2 OR S3 OR S4 OR S5 OR S6 OR S7 OR S8 OR S9 OR S10 OR S11 OR S12 OR S13 |  |  | S51 | TI (trial or effect* or impact* or  intervention* or before N5 after or pre N5 post or ((pretest or "pre test") and (posttest or "post test")) or quasiexperiment* or quasi W0 experiment* or pseudo experiment* or pseudoexperiment* or evaluat* or "time  series" or time W0 point* or repeated W0 measur*) OR AB (trial or effect* or impact* or intervention* or before N5 after or pre N5 post or ((pretest or "pre test") and (posttest or "post test")) or quasiexperiment* or quasi W0 experiment* or pseudo experiment* or pseudoexperiment* or evaluat* or "time  series" or time W0 point* or repeated W0 measur*) |  |  |
|  |  | S15 | treat* or therap* |  |  | S52 | S38 OR S39 OR S40 OR S41 OR S42 OR S43 OR S44 OR S45 OR S46 OR S47 OR S48 OR S49 OR S50 OR S51 |  |  |
|  |  | S16 | (MH "Protocols+") |  |  |  |  |  |  |
|  |  | S17 | S15 AND S16 |  |  |  |  |  |  |
|  |  | S18 | (MH "Practice Guidelines") |  |  |  |  |  |  |
|  |  | S19 | implement* or pathway or  protocol? |  |  |  |  |  |  |
|  |  | S20 | S18 AND S19 |  |  |  |  |  |  |
|  |  | S21 | guideline? N1 (implement* or pathway or protocol?) |  |  |  |  |  |  |
|  |  | S22 | S20 OR S21 |  |  |  |  |  |  |
|  |  | S23 | S14 OR S17 OR S22 |  |  |  |  |  |  |
|  |  |  | | | | | |  |  |
|  |  | S36 | S23 AND S35 | | |  |  |  |  |
|  |  | S37 | S1 OR S36 |  |  |  |  |  |  |
|  |  |  | | | | | |  |  |
|  |  | **S53** | **S37 AND S52** | | | | |  | **498** |
|  | 2019/07/15 | **S53** | **S37 AND S52** | | | | | **Extended published date to 2019/12/31** | **1,395** |
| **Cochrane**  **Library** | 2017/12/19 | #1 | (clinical near/2 pathway?):ti | #24 | [mh "primary health care"] |  | / | **Publication year from 2007 to 2017** |  |
|  |  | #2 | [mh "critical pathways"] | #25 | [mh "family practice"] |  |  |  |  |
|  |  | #3 | ((clinical or critical) near/1 (pathway? or path?)):ti,ab | #26 | [mh "physicians, primary care"] |  |  |  |  |
|  |  | #4 | ((care near/2 algorithm?) or (clinical next algorithm?)):ti,ab | #27 | [mh "general practice"] |  |  |  |  |
|  |  | #5 | (care near/1 pathway*):ti,ab | #28 | [mh "physicians, family"] |  |  |  |  |
|  |  | #6 | (treatment near/3  algorithm*):ti,ab | #29 | [mh "general practitioners"] |  |  |  |  |
|  |  | #7 | ((management next protocol?) or  (treatment next protocol?)):ti,ab | #30 | [mh "outpatient clinics, hospital"] |  |  |  |  |
|  |  | #8 | (care near/1 (plan? or map?)):ti,ab | #31 | [mh "ambulatory care"] |  |  |  |  |
|  |  | #9 | (protocol? near/1 (nursing or directed or guided)):ti,ab | #32 | [mh "ambulatory care facilities"] |  |  |  |  |
|  |  | #10 | ((local or locally) near/2 adapt* near/5 guideline?):ti,ab | #33 | [mh "community health services"] |  |  |  |  |
|  |  | #11 | ((treatment next model?) near/10  standardi*):ti,ab | #34 | [mh "community health centers"] |  |  |  |  |
|  |  | #12 | (standardi* near/3  protocol?):ti,ab | #35 | ((primary or communit*) near/5 (care or health*)):ti,ab |  |  |  |  |
|  |  | #13 | (systematic next  detection):ti,ab | #36 | (family next practi* or family next doctor* or family next physician* or gp* or general next practi*):ti,ab |  |  |  |  |
|  |  | #14 | (or #2-#13) | #37 | ((outpatient? or ambulatory) near/2 (care or healthcare or clinic? or service? or  facilit*)):ti,ab |  |  |  |  |
|  |  | #15 | [mh "clinical protocols"] | #38 | {or #24-#37} |  |  |  |  |
|  |  | #16 | (treat* or therap*):ti,ab |  |  |  |  |  |  |
|  |  | #17 | #15 and #16 |  |  |  |  |  |  |
|  |  | #18 | [mh "practice guidelines as topic"] |  |  |  |  |  |  |
|  |  | #19 | (implement* or pathway or  protocol?):ti,ab |  |  |  |  |  |  |
|  |  | #20 | #18 and #19 |  |  |  |  |  |  |
|  |  | #21 | (guideline? near/1  (implement* or pathway or  protocol?)):ti,ab |  |  |  |  |  |  |
|  |  | #22 | #20 or #21 |  |  |  |  |  |  |
|  |  | #23 | #14 or #17 or #22 |  |  |  |  |  |  |
|  |  |  | | | | | |  |  |
|  |  | **#39** | **#1 or (#23 and #38)** | | |  |  |  | **980** |
|  | 2019/07/15 | **#39** | **#1 or (#23 and #38)** | | |  |  | **Extended publication year to 2019** | **2,179** |
