# Supplementary material for: Barriers to and facilitators of the implementation of multi-disciplinary care pathways in primary care: a systematic review
Source: BMC Fam Pract. 2020 Jun 19;21:113. doi: 10.1186/s12875-020-01179-w (PMC7305630; doi:10.1186/s12875-020-01179-w)
Supplement: Supplementary file 2 — Additional file 2. Methodological quality of included main project reports. [file 12875_2020_1179_MOESM2_ESM.docx]

**Additional file 2** Methodological quality of included main project reports

| **AZAD ET AL., 2008 [30]** | | |
| --- | --- | --- |
| **Bias** | **Authors' judgement** | **Support for judgement** |
| Random sequence generation  (selection bias) | Low risk | Quote: "Following recruitment, patients were randomly assigned (via computer-generated randomisation) or either usual care (control group), or the clinical pathway program (treatment group)." |
| Allocation concealment  (selection bias) | Unclear risk | Comment: Insufficient information. |
| Blinding of participants and personnel (performance bias) | Unclear risk | Comment: Insufficient information. |
| Blinding of outcome assessment (detection bias) | Low risk | Quote: "Data was collected, by an assessor, blinded from both treatment and control groups at four points in time." |
| Incomplete outcome data  (attrition bias) | Low risk | Comment: More participants were lost to follow-up in the control group than in the intervention group (IG: 0%; CG: 15%) with reasons insufficient stated.  Quote: “Results were analysed via an intent-to-treat approach.” |
| Selective reporting  (reporting bias) | Unclear risk | Comment: No indication to study protocol. |
| Other bias | High risk | Quote: "Initially, the planned sample size was 100 participants in each group. ... Due to poor recruitment and limited resources it was not possible to recruit 200 participants." |
| **BLEIJENBERG ET AL., 2016a [32]** | | |
| **Bias** | **Authors' judgement** | **Support for judgement** |
| Random sequence generation  (selection bias) | Low risk | Quote: "... using a computer-generated random allocation sequence with the aim of an allocation ratio at the individual level of 1:1:1." |
| Allocation concealment  (selection bias) | Unclear risk | Comment: Insufficient information. |
| Blinding of participants and personnel (performance bias) | Unclear risk | Quote: "Individuals were not aware of the intervention arm that they were allocated to but were fully informed at the end of the study."  "This was a single-blind … trial." "Because GPs in the control group were not blinded, they may have upgraded their usual care, resulting in diminished effectiveness of the intervention." |
| Blinding of outcome assessment (detection bias) | Unclear risk | Quote: "The investigators were not blinded for logistical reasons."  Comment: Investigator = outcome assessor? |
| Incomplete outcome data  (attrition bias) | Low risk | Comment: Missing outcome data balanced in numbers across groups, with similar reasons for missing data across groups.  Quote: "An intention-to-treat analysis was performed to detect differences between the intervention groups and the control group." |
| Selective reporting  (reporting bias) | High risk | Comment: Indication to study protocol, but differences descripted. |
| Other bias | Unclear risk | Comment: The authors listed a few potential influencing factors, but it is not clear how far these may skew the results. |
| Cluster randomized trials | High risk | Quote: "Eligible individuals were identiﬁed after randomization." "This was a single-blind … trial."  Comment: Baseline comparability of clusters was reported; there was statistical adjustment for baseline characteristics; missing outcomes for clusters or within clusters were not reported (and 4 general practices dropped out after randomisation but before recruiting participants); accounted for the clustered design in the analysis; results comparable with individually-randomised trials. |
| **HARRIS ET AL, 2015 [36]** | | |
| **Bias** | **Authors' judgement** | **Support for judgement** |
| Random sequence generation  (selection bias) | Low risk | Quote: "… using the Nottingham Clinical Trials Unit internet randomisation service. … Block randomisation was used within practice with random sized blocks, varying between 4 and 6, and 1:1 allocation ratio, to ensure group balance." |
| Allocation concealment  (selection bias) | Unclear risk | Comment: Insufficient information. |
| Blinding of participants and personnel (performance bias) | High risk | Quote: "Participants were informed by telephone of their group allocation.”  Comment: Insufficient information about blinding of personnel. |
| Blinding of outcome assessment (detection bias) | High risk | Quote: "Assessors were not blinded to group status." |
| Incomplete outcome data  (attrition bias) | Low risk | Comment: Missing outcome data balanced in numbers across groups, with similar reasons for missing data across groups. |
| Selective reporting  (reporting bias) | Low risk | Comment: Indication to study protocol and all pre-defined outcomes measured. |
| Other bias | Unclear risk | - |
| Cluster randomized trials | Low risk | Comment: Insufficient information about the time point of cluster randomization; missing outcomes for clusters or within clusters were reported; accounted for the clustered design in the analysis; results comparable with individually-randomised trials.  Quote: "Baseline characteristics were similar between randomised groups except that participants in the intervention group were on averages lightly older than those in the control group, more likely to have left school younger, be overweight or obese, more likely to have chronic diseases and disability, and a slightly lower baseline step-count." |
| **MELIS ET AL., 2008 [37]** | | |
| **Bias** | **Authors' judgement** | **Support for judgement** |
| Random sequence generation  (selection bias) | Unclear risk | Quote: "Pseudocluster randomization randomized physicians in two groups."  Comment: Insufficient information about the random sequence generation process. |
| Allocation concealment  (selection bias) | Unclear risk | Comment: Insufficient information. |
| Blinding of participants and personnel (performance bias) | Unclear risk | Quote: "The physicians were not informed as to which group they were in." "… the majority of physicians were not aware of the allocated randomization proportions."  Comment: Insufficient information. |
| Blinding of outcome assessment (detection bias) | Low risk | Quote: "This study was observer blind." |
| Incomplete outcome data  (attrition bias) | Unclear risk | Quote: "The total dropout rate in our study was 7% at 3 months and 13% at 6 months, and was similar in both groups. ... The total dropout in our study was fairly high, but was similar in both groups and was as expected when taking into account the frailty of the population. Dropouts occurred mainly because patients (or their caregivers) felt participation in follow-up visits for effect measurement was too burdensome while it provided no further benefit."  "Modified intention-to-treat analyses focused on differences between treatment arms in functional abilities and mental wellbeing." |
| Selective reporting  (reporting bias) | Low risk | Comment: Indication to study protocol and all pre-defined outcomes measured. |
| Other bias | Unclear risk | - |
| Cluster randomized trials | Low risk | Quote: "We used a twostep pseudocluster randomization procedure … randomized physicians in two groups: group H (high) and group L (low). The participants recruited through physicians of group H were then randomized in an 80/20 ratio to DGIP and usual care, respectively; in group L this ratio was reversed: 20% DGIP and 80% usual care. … In the second step of the pseudocluster randomization procedure, minimization was used to equally distribute participants … This approach has the advantage, especially in small trials, that there will be only minor differences between groups in those variables used in the allocation process."  "Baseline characteristics … showed no signiﬁcant differences between study groups." "Our control group was smaller than the intervention group, but it is unlikely that lack of allocation concealment has caused the difference; the majority of physicians were not aware of the allocated randomization proportions. Patients were comparable at baseline as well, giving no indication of selection bias."  Comment: Missing outcomes for clusters or within clusters were not reported; accounted for the clustered design in the analysis; results comparable with individually-randomised trials. |
| **METZELTHIN ET AL., 2013b [39]** | | |
| **Bias** | **Authors' judgement** | **Support for judgement** |
| Random sequence generation  (selection bias) | Low risk | Quote: "... used a computer generated randomisation list." |
| Allocation concealment  (selection bias) | Unclear risk | Comment: Insufficient information. |
| Blinding of participants and personnel (performance bias) | High risk | Quote: "Older people with healthcare professionals were aware of the allocated arm (intervention or control)." |
| Blinding of outcome assessment (detection bias) | Low risk | Quote: "Outcome assessors were kept blinded to the allocation." |
| Incomplete outcome data  (attrition bias) | High risk | Quote: "... significantly more participants were lost to follow-up in the intervention group than in the control group (26% v 17%). ... older people in the intervention group were significantly more frail and disabled than those in the control group, which might have affected the completion rate."  "We analysed the primary and secondary outcomes, measured at the level of the patient, according to the intention to treat principle." |
| Selective reporting  (reporting bias) | Low risk | Comment: Indication to study protocol and all pre-defined outcomes measured. |
| Other bias | Unclear risk | - |
| Cluster randomized trials | High risk | Quote: "… significant baseline differences existed between the intervention and control groups with regard to frailty and disability, and the sample size distribution was skewed."  Comment: Individuals were recruited to the trial after the clusters were randomised and personnel recruiting participants were not blinded to cluster; there was statistical adjustment for baseline characteristics; missing outcomes for clusters or within clusters were not reported; accounted for the clustered design in the analysis; results comparable with individually-randomised trials. |
| **VAN BRUGGEN ET AL., 2008 [41]** | | |
| **Bias** | **Authors' judgement** | **Support for judgement** |
| Random sequence generation  (selection bias) | Low risk | Quote: "… using a random number table to ensure equal numbers of practices in each group." |
| Allocation concealment  (selection bias) | Low risk | Quote: "An independent researcher then carried out a restricted randomization procedure." |
| Blinding of participants and personnel (performance bias) | Unclear risk | Comment: Insufficient information about blinding of participants.  Quote: "Control group practices were asked to continue the care for people with diabetes in line with the national guidelines." |
| Blinding of outcome assessment (detection bias) | Unclear risk | Comment: Insufficient information. |
| Incomplete outcome data  (attrition bias) | Low risk | Comment: Missing outcome data balanced in numbers across groups, with similar reasons for missing data across groups.  Quote: "All analyses were by intention to treat." |
| Selective reporting  (reporting bias) | Unclear risk | Comment: Insufficient information. No indication to study protocol. |
| Other bias | Unclear risk | - |
| Cluster randomized trials | Unclear risk | Comment: Insufficient information about the time point of cluster randomization; there was statistical adjustment for baseline characteristics; missing outcomes for clusters or within clusters were not reported; accounted for the clustered design in the analysis; results comparable with individually-randomised trials.  Quote: "Except for education and the presence of macrovascular complications, patients’ characteristics were highly comparable across study groups." |
| **WELDAM ET AL., 2017a [42]** | | |
| **Bias** | **Authors' judgement** | **Support for judgement** |
| Random sequence generation  (selection bias) | Low risk | Quote: "... using a computer-generated randomisation programme with block randomisation, developed by an independent data manager from the University Medical Center Utrecht." |
| Allocation concealment  (selection bias) | Unclear risk | Comment: Insufficient information. |
| Blinding of participants and personnel (performance bias) | High risk | Quote: "... participating nurses and patients could not be blinded to allocation." |
| Blinding of outcome assessment (detection bias) | Unclear risk | Comment: Insufficient information. |
| Incomplete outcome data  (attrition bias) | Low risk | Comment: Missing outcome data balanced in numbers across groups (IG: 17%, CG: 14%), with similar reasons for missing data across groups.  Quote: "The primary effectiveness analysis was an intention-to-treat analysis." |
| Selective reporting  (reporting bias) | Unclear risk | Comment: No indication to study protocol. |
| Other bias | High risk | Quote: "... we did not succeed in including the number of patients calculated in the power calculation." |
| Cluster randomized trials | Low risk | Quote: "Randomisation was performed at the level of the primary care practices/home care services before the inclusion of patients." "There were no significant differences between the two groups at baseline, meaning that the two trial arms were well balanced on all variables at patient level."  Comment: Missing outcomes for clusters or within clusters were not reported; accounted for the clustered design in the analysis; results comparable with individually-randomised trials. |
