# Supplementary figures and images for: Barriers to and facilitators of the implementation of multi-disciplinary care pathways in primary care: a systematic review
Source: BMC Fam Pract. 2020 Jun 19;21:113. doi: 10.1186/s12875-020-01179-w (PMC7305630; doi:10.1186/s12875-020-01179-w)

**Additional file 3** Risk of bias summary of RCTs and cRCTs (designed by using RevMan [44])


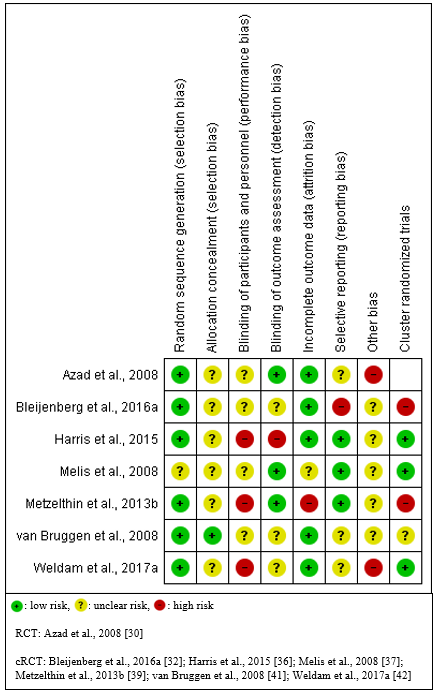

Supplement: Supplementary file 3 — Additional file 3. Risk of bias summary of RCTs and cRCTs (designed by using RevMan [44]). [file 12875_2020_1179_MOESM3_ESM.docx]
