# Supplementary material for: Barriers to and facilitators of the implementation of multi-disciplinary care pathways in primary care: a systematic review
Source: BMC Fam Pract. 2020 Jun 19;21:113. doi: 10.1186/s12875-020-01179-w (PMC7305630; doi:10.1186/s12875-020-01179-w)
Supplement: Supplementary file 4 — Additional file 4. Overview of critical appraisal tools used for different study designs. [file 12875_2020_1179_MOESM4_ESM.docx]

**Additional file 4** Overview of critical appraisal tools used for different study designs

| **Study design*** | **Critical appraisal tool** | **Used quality assessment criteria/questions** |
| --- | --- | --- |
| Randomized  controlled trials | Cochrane Collaboration’s tool for assessing risk of Bias [45]  (RoB) | - random sequence generation (selection bias) - allocation concealment (selection bias) - blinding of participants and personnel (performance bias) - blinding of outcome assessment (detection bias) - incomplete outcome data (attrition bias) - selective reporting (reporting bias) - other bias   In cluster randomized trials, we also considered particular biases:   - recruitment bias - baseline imbalance - loss of clusters - incorrect analysis - comparability with individually randomized trials |
| Qualitative studies | Critical Appraisal Skills Programme [47]  (CASP) | - Was there a clear statement of the aims of the research? - Is a qualitative methodology appropriate? - Was the research design appropriate to address the aims of the research? - Was the recruitment strategy appropriate to the aims of the research? - Was the data collected in a way that addressed the research issue? - Has the relationship between researcher and participants been adequately   considered?   - Have ethical issues been taken into consideration? - Was the data analysis sufficiently rigorous? - Is there a clear statement of findings? |
| Mixed-methods studies | Mixed Methods  Appraisal Tool [48]  (MMAT) | Screening Questions (for all types)   - Are there clear qualitative and quantitative research questions (or objectives), or a clear mixed methods question (or objective)? - Do the collected data allow to address the research question (objective)? E.g., consider whether the follow-up period is long enough for the outcome to occur (for longitudinal studies or study components).   Qualitative   - Are the sources of qualitative data (archives, documents, informants, observations) relevant to address the research question (objective)? - Is the process for analyzing qualitative data relevant to address the research question (objective)? - Is appropriate consideration given to how findings relate to the context, e.g., the setting, in which the data were collected? - Is appropriate consideration given to how findings relate to researchers’ influence, e.g., through their interactions with participants?   Quantitative descriptive   - Is the sampling strategy relevant to address the quantitative research question (quantitative aspect of the mixed methods question)? - Is the sample representative of the population understudy? - Are measurements appropriate (clear origin, or validity known, or standard instrument)? - Is there an acceptable response rate (60% or above)?   Mixed methods   - Is the mixed methods research design relevant to address the qualitative and quantitative research questions (or objectives), or the qualitative and quantitative aspects of the mixed methods question (or objective)? - Is the integration of qualitative and quantitative data (or results) relevant to address the research question (objective)? - Is appropriate consideration given to the limitations associated with this integration, e.g., the divergence of qualitative and quantitative data (or results) in a triangulation design? |

*Since we finally did not include any other studies than RCTs (*main project reports*), we refrained from listing details of other critical appraisal tools.
