# Supplementary material for: Barriers to and facilitators of the implementation of multi-disciplinary care pathways in primary care: a systematic review
Source: BMC Fam Pract. 2020 Jun 19;21:113. doi: 10.1186/s12875-020-01179-w (PMC7305630; doi:10.1186/s12875-020-01179-w)
Supplement: Supplementary file 5 — Additional file 5. Main components of the interventions reported in the included main project reports. [file 12875_2020_1179_MOESM5_ESM.docx]

**Additional file 5** Main components of the interventions reported in the included main project reports

| **Source,**  **year** | **Development and piloting** | | | **Components of the intervention: recipient** | | | | **Components of**  **intervention:**  **provider** |
| --- | --- | --- | --- | --- | --- | --- | --- | --- |
|  | Evidence-based | Involvement of clinicians | Previous  feasibility/ pilot study | Assessment | Individually  tailored  treatment | Locally adapted  recommendations | Regular  evaluation/  monitoring | Training  activities |
| Azad et al., 2008 [30] | ✓ | not reported | ✓ | ✓ | ✓ | X | ✓ | ✓ |
| Bleijenberg et al., 2016a [32] | ✓ | ✓ | ✓ | ✓ | ✓ | X | ✓ | ✓ |
| Harris et al., 2015 [36] | ✓ | not reported | not reported | not reported | ✓ | X | ✓ | ✓ |
| Melis et al., 2008 [37] | ✓ | not reported | ✓ | ✓ | ✓ | X | ✓ | not reported |
| Metzelthin et al., 2013b [39] | ✓ | ✓ | ✓ | ✓ | ✓ | X | ✓ | ✓ |
| van Bruggen et al. 2008 [41] | ✓ | ✓ | not reported | not reported | not reported | ✓ | not reported | ✓ |
| Weldam et al., 2017b [43] | ✓ | ✓ | not reported | ✓ | ✓ | X | ✓ | ✓ |

✓=Yes; X=NO
