# Supplementary material for: Barriers to and facilitators of the implementation of multi-disciplinary care pathways in primary care: a systematic review
Source: BMC Fam Pract. 2020 Jun 19;21:113. doi: 10.1186/s12875-020-01179-w (PMC7305630; doi:10.1186/s12875-020-01179-w)
Supplement: Supplementary file 6 — Additional file 6. Quality assessment results of aspects of the qualitative studies (CASP Checklist). [file 12875_2020_1179_MOESM6_ESM.docx]

**Additional file 6** Quality assessment results of aspects of the qualitative studies (CASP Checklist)

| **Quality assessment question** | Bleijenberg et al., 2015 [34] | Harris et al.,  2015 [36] | van Bruggen et  al., 2008 [41] |
| --- | --- | --- | --- |
| Was there a clear statement of the aims of the research? | ✓ | ✓ | can’t tell |
| Is a qualitative methodology appropriate? | ✓ | ✓ | ✓ |
| Was the research design appropriate to address the aims of the research? | ✓ | ✓ | can’t tell |
| Was the recruitment strategy appropriate to the aims of the research? | can’t tell | can’t tell | can’t tell |
| Was the data collected in a way that addressed the research issue? | ✓ | ✓ | can’t tell |
| Has the relationship between researcher and participants been  adequately considered? | can’t tell | can’t tell | can’t tell |
| Have ethical issues been taken into consideration? | can’t tell | ✓ | ✓ |
| Was the data analysis sufficiently rigorous? | ✓ | can’t tell | can’t tell |
| Is there a clear statement of findings? | ✓ | can’t tell | ✓ |

✓=Yes; [X=NO]
