# Supplementary material for: Barriers to and facilitators of the implementation of multi-disciplinary care pathways in primary care: a systematic review
Source: BMC Fam Pract. 2020 Jun 19;21:113. doi: 10.1186/s12875-020-01179-w (PMC7305630; doi:10.1186/s12875-020-01179-w)
Supplement: Supplementary file 7 — Additional file 7. Quality assessment results of aspects of the mixed-method studies (MMAT). [file 12875_2020_1179_MOESM7_ESM.docx]

**Additional file 7**. Quality assessment results of aspects of the mixed-method studies (MMAT)

| **Quality assessment question** | Bleijenberg et al., 2013b [33] | Bleijenberg et al., 2016b [35] | Weldam et al., 2017b [43] | Metzelthin et al., 2013a [40] |
| --- | --- | --- | --- | --- |
| **Screening Questions (for all types)** |  |  |  |  |
| Are there clear qualitative and quantitative research questions (or objectives), or a clear mixed methods question (or objective)? | ✓ | ✓ | ✓ | ✓ |
| Do the collected data allow address the research question (objective)? E.g., consider whether the follow-up period is long enough for the outcome to occur (for longitudinal studies or study components). | can’t tell | ✓ | ✓ | ✓ |
| **Qualitative** |  |  |  |  |
| Are the sources of qualitative data (archives, documents, informants, observations) relevant to address the research question (objective)? | ✓ | ✓ | ✓ | ✓ |
| Is the process for analyzing qualitative data relevant to address the research question (objective)? | ✓ | ✓ | ✓ | ✓ |
| Is appropriate consideration given to how findings relate to the context, e.g., the setting, in which the data were collected? | ✓ | ✓ | ✓ | ✓ |
| Is appropriate consideration given to how findings relate to researchers’ influence, e.g., through their interactions with participants? | can’t tell | can’t tell | can’t tell | can’t tell |
| **Quantitative descriptive** |  |  |  |  |
| Is the sampling strategy relevant to address the quantitative research question (quantitative aspect of the mixed methods question)? | ✓ | ✓ | ✓ | ✓ |
| Is the sample representative of the population understudy? | ✓ | ✓ | ✓ | ✓ |
| Are measurements appropriate (clear origin, or validity known, or standard instrument)? | can’t tell | can’t tell | can’t tell | can’t tell |
| Is there an acceptable response rate (60% or above)? | ✓ | ✓ | ✓ | ✓ |
| **Mixed methods** |  |  |  |  |
| Is the mixed methods research design relevant to address the qualitative and quantitative research questions (or objectives), or the qualitative and quantitative aspects of the mixed methods question (or objective)? | ✓ | ✓ | ✓ | ✓ |
| Is the integration of qualitative and quantitative data (or results) relevant to address the research question (objective)? | ✓ | ✓ | ✓ | ✓ |
| Is appropriate consideration given to the limitations associated with this integration, e.g., the divergence of qualitative and quantitative data (or results) in a triangulation design? | ✓ | can’t tell | can’t tell | ✓ |

✓=Yes; [X=NO]
